# Supplementary material for: Adaptive exponential weighted composite sliding mode-based direct yaw moment control for four-wheel independently actuated autonomous vehicles
Source: PLoS One. 2025 Aug 21;20(8):e0330474. doi: 10.1371/journal.pone.0330474 (PMC12370079; doi:10.1371/journal.pone.0330474)
Supplement: Attachment — Submitted filename: Response to Reviewers.docx [file pone.0330474.s001.docx]

**Original Manuscript ID:** PONE-D-25-19826

**Original Article Title: “Adaptive exponential weighted composite sliding mode-based direct yaw moment control for four-wheel independently actuated autonomous vehicles**”

**To:** PLOS ONE

**Re:** Response to reviewers

Dear Editor,

First and foremost, we sincerely thank you for giving us the opportunity to revise our manuscript and to address the reviewers' comments. We dedicated nearly a month to carefully addressing all points raised by the reviewers. This involved comprehensive literature reviews, detailed technical revisions, and extensive discussions among the authors. The manuscript has undergone significant modification, and we deeply appreciate the reviewers' highly professional and insightful expertise, which has significantly enhanced the quality of our work.

We will be submitting: (a) Our point-by-point response to all comments (contained in the "Response to Reviewers" document below); (b) An updated manuscript with changes highlighted (using yellow highlighting) ("Revised Manuscript with Track Changes"); and (c) A clean version of the updated manuscript without highlighting ("Manuscript").

Should any further improvements be required, we warmly welcome additional feedback to ensure the manuscript meets the journal’s standards.

Thank you once again for your guidance and support throughout this process.

Best regards,

<Zhongzhi Tong> et al.

School of Mechanical Engineering

Nanjing University of Science and Technology

Nanjing, Jiangsu, China

2025/06/01

**Additional Editor Comments#1:** The background should be updated to cite recent research on direct yaw moment control.

**Author response and action:** We sincerely appreciate your valuable suggestion to strengthen the background by incorporating recent research on direct yaw moment control. This feedback significantly enhances the manuscript's relevance to current advancements in the field. Accordingly, we have conducted a thorough review of the most recent high-quality literature and have updated the background section to include the following pertinent references:

“A Direct Yaw Moment Control Framework Through Robust T-S Fuzzy Approach Considering Vehicle Stability Margin. IEEEASME Trans Mechatron. 2024 Feb;29(1):166–78”, “A Robust Dynamic Game-Based Control Framework for Integrated Torque Vectoring and Active Front-Wheel Steering System. IEEE Trans Intell Transp Syst. 2023 Jul;24(7):7328–41”, and “An Energy-Oriented Torque-Vector Control Framework for Distributed Drive Electric Vehicles. IEEE Trans Transp Electrification. 2023 Sep;9(3):4014–31”.

We believe these additions strengthen the theoretical foundation of our work. We are grateful for this insightful comment, which has enhanced the manuscript's scholarly quality.

**Reviewer#1, Concern #1:** When elaborating on its innovative points, the paper not only provides a detailed description of the control method itself, but also further emphasizes its unique advantages in yaw control.

**Author response and action:** Thank you for your recognition of our elaboration on the innovative aspects. Following your insightful suggestions, we have implemented thorough revisions that have substantially enhanced the overall quality of the paper. We greatly appreciate your valuable guidance and expertise.

**Reviewer#1, Concern #2:** The authors listed many literatures on relevant research results, but what are their advantages and shortcomings? The literature review should point out the corresponding shortcomings to support your research.

**Author response and action:** We sincerely appreciate your insightful suggestion. We fully concur that a comprehensive literature review should not only summarize existing research but also critically evaluate their strengths and limitations to contextualize our contribution. In response to this feedback, we have meticulously revised the Introduction section (pages 3–5) to incorporate a nuanced discussion of the advantages and shortcomings of the cited studies. Once again, we sincerely appreciate your professional and insightful feedback.

**Reviewer#1, Concern # 3:** The paper uses a 2-DOF vehicle model to calculate the expected yaw rate for the control of a four-wheel independent drive vehicle. When allocating the yaw moment, in addition to constraining energy, is it necessary to further consider other constraints?

**Author response and action:** We sincerely appreciate your professional and insightful feedback. In response, we have supplemented the 'Lower Allocation Design' section with discussions of friction circle constraints and actuator saturation constraints to enhance the robustness of our methodology. Furthermore, we conducted comprehensive simulations using four distinct test cases to rigorously validate these enhancements. We are grateful for your constructive feedback, which has significantly improved the technical completeness and academic rigor of our work.

**Reviewer#1, Concern # 4:** Table 1 lists the vehicle parameters and controller related parameters. Is it reasonable to set the constraint on the maximum steering angle to 0.5? Is there sufficient theoretical basis or practical application value for this?

**Author response and action:** We sincerely appreciate your meticulous and professional feedback. In response to your inquiry, we consulted relevant vehicle specifications and literature, which indicate that typical maximum steering angles for passenger vehicles range between 30°-40°. Based on this practical benchmark, we have revised our steering angle constraint from 0.5 rad（28.65°）to 40*π/180 rad（40°）n the updated manuscript. Additionally, we conducted a comprehensive sensitivity analysis of key parameters and validated these adjustments through four rigorous simulation scenarios. We are deeply grateful for your insightful guidance, which has substantially elevated the technical accuracy and practical relevance of our work.

**Reviewer#1, Concern # 5:** Does formula 41 have the problem of non-uniqueness of solutions when solving the yaw moments of the four wheels? For example, could this situation occur when the vehicle parameters are set differently? Therefore, further analyzing the robustness of the control system and verifying it through simulation results are suggested.

**Author response and action:** We sincerely appreciate your rigorous and professional feedback. In response to your concern regarding the non-uniqueness of solutions for Formula 41, we have supplemented a theoretical analysis of uniqueness on page 19 of the manuscript. Additionally, we expanded our simulations to include four distinct test cases. The simulation results not only demonstrate the rationality of our allocation design but also reflect the robustness of the control system. This revision process has greatly benefited us, and we once again sincerely thank you for your invaluable assistance and guidance.

**Reviewer#1, Concern # 6:** How is the parameter τ in formula 26 set during the simulation experiment?

**Author response and action:** We sincerely apologize for the omission of the parameter τ in the previous simulation parameter table. This parameter has now been updated in the revised table. The configuration of τ incorporates the influence of the front wheel steering angle through a piecewise setup. We are once again deeply grateful for your meticulous and professional review, which has significantly enhanced the rigor and accuracy of our work.

**Reviewer#1, Concern # 7:** There is an inaccuracy in the description of Figure 8 in the paper. It is recommended to recheck and correct it.

**Author response and action:** We sincerely apologize for the oversight in the original manuscript, where the labels of two figures were accidentally swapped. We have corrected this error and thoroughly proofread the entire manuscript to prevent similar issues, ensuring the accuracy of all data and figures. We once again sincerely appreciate your professional and rigorous review, which has greatly enhanced the precision and academic rigor of our paper.

**Reviewer#1, Concern # 8:** More latest sliding mode control methods are suggested to be introduced to compare in the simulation experiment.

**Author response and action:** We sincerely appreciate your insightful and professional suggestion. To address your recommendation, we conducted an extensive literature review and implemented the state-of-the-art Nonsingular Terminal Sliding Mode Control (NTSMC) strategy, which represents a cutting-edge advancement in sliding mode control theory. This advanced methodology was incorporated into our simulation framework and rigorously evaluated against four distinct test cases. We are deeply grateful for your recommendation, which has significantly elevated the academic rigor and innovation of our research.

**Reviewer#1, Concern # 9:** The English of the article should be thoroughly revised.

**Author response and action:** We have conducted a comprehensive revision of the English language throughout the entire manuscript. We sincerely appreciate your critical feedback, which has been instrumental in elevating the overall professionalism and readability of our work.

**Reviewer#2, Concern # 1:** Incorporate experimental validation or discuss simulation limitations in detail.

**Author response and action:** We sincerely appreciate your professional feedback. To address the limitations of our simulation, we have supplemented additional simulation experiments, designing four comparative simulation scenarios under two representative operating conditions. Furthermore, we incorporated the advanced Nonsingular Terminal Sliding Mode Control (NTSMC) strategy for rigorous comparative validation. Your insightful recommendations have significantly enhanced the rigor and comprehensiveness of our work. We extend our heartfelt gratitude once again for your invaluable guidance.

**Reviewer#2, Concern # 2:** Expand comparisons to include other advanced control methods.

**Author response and action:** Following your valuable suggestion, we conducted an extensive literature review and replicated the advanced Nonsingular Terminal Sliding Mode Control (NTSMC) strategy, incorporating it into our simulation comparison framework. We designed four distinct simulation scenarios to rigorously evaluate its performance against existing methods. This revision has substantially elevated the technical depth and quality of our work. We sincerely appreciate your expert guidance, which has been instrumental in refining our research.

**Reviewer#2, Concern # 3:** Conduct sensitivity analysis for key parameters and test under varied conditions.

**Author response and action:** We sincerely appreciate your insightful recommendation. In response, we conducted a comprehensive sensitivity analysis of critical parameters, employing sideslip angle Mean Absolute Error (MAE) and yaw rate MAE as quantitative performance indicators. This analysis enabled us to identify optimal parameter configurations and refine our simulation framework accordingly. The updated Simulation Parameters table now reflects these optimizations, and we have validated their robustness through four comparative simulation scenarios under varied operating conditions. The results conclusively demonstrate the enhanced stability and adaptability of our proposed control system. We are deeply grateful for your expertise, which has significantly strengthened the technical rigor and academic credibility of our work.

**Reviewer#2, Concern # 4:** Explicitly address limitations and improve clarity for a broader audience.

**Author response and action:** We sincerely appreciate your professional feedback. To address the limitations of our simulation, we have supplemented additional simulation experiments, designing four comparative simulation scenarios under two representative operating conditions. Furthermore, we incorporated the advanced Nonsingular Terminal Sliding Mode Control (NTSMC) strategy for rigorous comparative validation. Your insightful recommendations have significantly enhanced the rigor and comprehensiveness of our work. We extend our heartfelt gratitude once again for your invaluable guidance.

**Reviewer#2, Concern # 5:** Ensure funding URLs are provided and references are formatted consistently.

**Author response and action:** We sincerely appreciate the insightful feedback regarding the funding information and reference formatting. The funding details have been successfully entered into the submission system. In accordance with the journal's formatting requirements, funding information should not be included in the manuscript itself; therefore, it has been omitted from the document. Concerning the reference formatting, we have utilized Zotero to generate the references, ensuring they are largely aligned with the journal's reference style guidelines. Once again, thank you for your valuable suggestions and guidance.
