# Supplementary material for: Adaptive exponential weighted composite sliding mode-based direct yaw moment control for four-wheel independently actuated autonomous vehicles
Source: PLoS One. 2025 Aug 21;20(8):e0330474. doi: 10.1371/journal.pone.0330474 (PMC12370079; doi:10.1371/journal.pone.0330474)
Supplement: Attachment — Submitted filename: Response_to_Reviewers_auresp_2.docx [file pone.0330474.s002.docx]

**Original Manuscript ID:** PONE-D-25-19826

**Original Article Title: “Adaptive exponential weighted composite sliding mode-based direct yaw moment control for four-wheel independently actuated autonomous vehicles**”

**To:** PLOS ONE

**Re:** Response to reviewers

Dear Editor,

We sincerely appreciate the opportunity to submit this revised manuscript. Recognizing the exceptional value of this resubmission invitation, we have approached the revision process with utmost rigor. Through comprehensive literature review, stringent technical refinements, and extensive collaborative deliberations, we have dedicated maximal effort to ensure the revisions meet the highest scholarly standards. We have now systematically addressed all reviewer comments. It is our privilege that you are considering this revised work, and we deeply value your continued support of this research.

We will be submitting: (a) Our point-by-point response to all comments (contained in the "Response to Reviewers" document below); (b) An updated manuscript with changes highlighted (using yellow highlighting) ("Revised Manuscript with Track Changes"); and (c) A clean version of the updated manuscript without highlighting ("Manuscript").

Should any further improvements be required, we warmly welcome additional feedback to ensure the manuscript meets the journal’s standards.

Thank you once again for your guidance and support throughout this process.

Best regards,

<Zhongzhi Tong> et al.

School of Mechanical Engineering

Nanjing University of Science and Technology

Nanjing, Jiangsu, China

2025/08/01

**Reviewer#3, Concern #1:** All performance claims (e.g., improved tracking accuracy, stability) are based on MATLAB/Simulink simulations. There is no demonstration that the proposed controller performs robustly in real-world scenarios with sensor noise, actuator delays, or unmodeled dynamics. If the experiments cannot be done, the authors may try to model more realistic dynamics into your model.

**Author response and action:** We sincerely thank you for your valuable professional suggestions. Based on your recommendations and to better approximate real driving scenarios, we have supplemented Gaussian white noise compliant with automotive-grade IMU specifications at the state feedback port of the controller to simulate sensor noise effects. Additionally, considering actuator delays, we have implemented actuator delay compensation code at both the upper and lower controller outputs. Finally, real-time friction circle verification has been added before torque limiting in the lower layer to ensure longitudinal demand forces do not exceed physical boundaries of the friction circle. We have systematically performed and re-analyzed simulation experiments, with detailed descriptions supplemented in Section V. We extend our profound gratitude for your expert guidance, which has significantly improved the engineering rigor and academic value of this research.

**Reviewer#3, Concern #2:** The AEWC-SMC controller is a variation of composite sliding mode control, and the DWMEA method is an adaptation of weighted minimum energy allocation. Prior works already address chattering suppression and allocation efficiency with similar strategies, as acknowledged in the literature review.

**Author response and action:** We sincerely thank you for your profound insights into the theoretical origins of the control strategy. Your accurate identification that AEWC-SMC and DWMEA are respectively grounded in the frameworks of Composite Sliding Mode Control and Weighted Energy Allocation has prompted us to more precisely articulate the differential innovations of this study:

1. While adopting the composite sliding mode structure, we achieve qualitative advancements through two innovative designs: First, the introduction of a nonlinear dynamic weighting factor enables sensitive response to large sideslip angle errors while maintaining linear accuracy in small-error regions, overcoming the response lag caused by fixed gains in traditional composite sliding mode structures. Subsequently, a condition-adaptive reaching law was designed, triggering fractional-order exponential switching via steering states to accelerate convergence during steering maneuvers while suppressing chattering in straight-line conditions.

2. While continuing the weighted energy allocation concept, the allocation solution employs a non-iterative closed-form method that achieves real-time allocation through KKT matrix inversion, and innovatively incorporates a dynamic weighting mechanism that first integrates the coupling effects of steering angle, vehicle speed, and vertical loads.

Accordingly, detailed supplementary explanations have been added to the opening paragraphs of Section III and Section IV, highlighted in yellow. Finally, we profoundly thank you for prompting clearer definition of our innovative boundaries. These invaluable suggestions have not only strengthened the precision of methodological exposition but also highlighted this study's original contributions to state-aware control architectures (AEWC-SMC) and real-time physical constraint resolution (DWMEA). Our team reiterates sincere gratitude for your suggestions and guidance.

**Reviewer#3, Concern # 3:** The abstract and conclusions claim “higher tracking accuracy, faster convergence speed, and enhanced handling stability,” but the improvements over advanced baselines (e.g., NTSMC) are marginal in several scenarios.

**Author response and action:** We sincerely thank you for your valuable suggestions. Based on Profes­sional Recommendation #1 mentioned earlier, we supplemented simulations of sensor noise and actuator delays to better approximate real-world condi­tions. On this basis, we rigorously revalidated the simulations. Results demonstrate that across four scenarios (Case1-Case4) under two extreme maneuvers (sine/hook maneuvers), the AEWC-SMC scheme consistently delivers non-marginal improvements with marked superiority. We therefore express heartfelt gratitude for your invaluable expertise. Your profound insights not only enabled us to identify and strengthen critical model components — such as sensor noise compensation and actuator delay handling — but also significantly enhanced the control scheme's robustness and verifiability under extreme conditions. These improvements fully underscore its practical advantages and innovative value in autonomous vehicle control domains. We anticipate incorporating your professional perspective into subsequent work to advance these findings toward engineering applications.

**Reviewer#3, Concern # 4:** The controller is not tested under more diverse or challenging conditions such as split-μ roads, sudden tire blowouts, actuator faults, or rapidly changing loads. Parameter sensitivity analysis is single-variable and does not explore interactions or robustness to parameter uncertainty.

**Author response and action:** We sincerely thank you for your in-depth review of this research work and your valuable suggestions! Your comments regarding the coverage of testing scenarios and parameter analysis methods have prompted us to more deeply reflect on the boundary conditions and optimization directions of this study. Below we will address your points by integrating research objectives, methodological selection rationale, and empirical validation outcomes:

I. **Explanation on Adequacy of Testing Scenarios**

The core research objective of this paper is to develop a real-time efficient Direct Yaw Moment Control (DYC) framework, focusing on solving the handling stability problem of four-wheel independently driven autonomous vehicles under typical extreme operating conditions (e.g., high lateral acceleration, low-adhesion road surfaces). For operating condition design, we followed standard testing specifications in the field of vehicle dynamics control (ISO 4138, SAE J266), selecting two types of extreme conditions: Sine Condition simulates transient responses during high-speed lane changes or obstacle avoidance; Fishhook Condition characterizes instability risks during emergency steering. Under each condition, we established multi-dimensional combined testing scenarios, with vehicle speeds covering medium-high ranges (22m/s and 33m/s), road adhesion coefficients including low-adhesion (μ=0.3) and high-adhesion (μ=0.8) conditions, and performance metrics simultaneously evaluating tracking accuracy (yaw rate MAE, sideslip angle MAE) and stability (phase-plane convergence regions). Simulation results also demonstrate that the proposed AEWC-SMC scheme significantly outperforms comparative algorithms across all testing scenarios. These results fully validate the controller's robustness under typical extreme operating conditions. Although more complex conditions such as split-μ roads are not included in this paper, the hierarchical control architecture established in this study (upper-layer adaptive sliding mode control + lower-layer dynamic weight allocation) lays the foundation for future extensions: the upper controller's nonlinear weighting factor can adaptively regulate sideslip error sensitivity, and the lower allocator's friction circle constraints inherently accommodate asymmetric adhesion conditions. We have also explicitly supplemented in Section VI that the next phase will involve hardware-in-the-loop validation, covering more complex fault conditions.

II. **Engineering Applicability of Parameter Sensitivity Analysis**

In this study, our core objective is to develop a novel hierarchical Direct Yaw Moment Control (DYC) scheme that integrates an upper-layer Adaptive Exponential Weighted Composite Sliding Mode Controller (AEWC-SMC) and a lower-layer Dynamic Weight Minimum Energy Allocation (DWMEA) method, aiming to enhance tracking accuracy, convergence speed, and handling stability for Four-Wheel Independently Actuated (FWIA) autonomous vehicles under extreme conditions. Single-variable sensitivity analysis is widely adopted in the preliminary stage of control system parameter tuning due to its conceptual clarity, computational efficiency, and ease of engineering implementation. We employ this method to rapidly identify parameters with the greatest impact on key system performance indicators (sideslip angle mean absolute error (MAE) and yaw rate MAE), thereby providing intuitive adjustment directions and laying the groundwork for preliminary controller gain tuning.

After parameter adjustments based on single-variable analysis results, the controller demonstrated significant performance improvements under both Sine Condition and Fishhook Condition. Specifically, in test cases combining multiple vehicle speeds and road adhesion coefficients (e.g., Case1: vx=22m/s, μ=0.3; Case4: vx=33m/s, μ=0.8), the optimized AEWC-SMC scheme outperformed both traditional Sliding Mode Control (SMC) and Nonsingular Terminal Sliding Mode Control (NTSMC) in sideslip angle tracking accuracy and yaw rate tracking accuracy. This effectively achieves the core objective of this study: enhancing vehicle dynamic response and stability under extreme operating conditions while ensuring high robustness.

We fully acknowledge, as you pointed out, that single-variable analysis indeed has limitations, especially when involving strongly coupled parameters or highly complex multi-variable interaction scenarios (e.g., simultaneously considering the dynamic weight influences of vehicle speed, steering angle, and vertical load), where its results may be insufficiently comprehensive. Conducting deeper multivariate analyses (such as Analysis of Variance (ANOVA), Sobol' sensitivity indices, or optimization-based global parameter searches) would be highly valuable research directions, helping to reveal nonlinear coupling mechanisms between parameters and optimize overall control performance. However, such analyses typically require significantly increased computational resources, experimental design complexity, and simulation time. Considering this study's primary focus on developing a real-time efficient control framework and validating its effectiveness under typical extreme conditions, along with current project resource constraints, conducting comprehensive analyses of this nature within this paper presents substantial challenges. Future work will explore these advanced analysis methods to further enhance controller adaptability and engineering applicability.

The simulation condition design and parameter analysis methods in this paper have achieved the preset research objective: verifying the superiority of the novel DYC framework under typical extreme operating conditions. Your suggestions have prompted us to more clearly recognize the need to add analysis of scenario extensibility feasibility in the discussion section, with parameter self-adaptation mechanisms becoming the next phase priority. We thank you again for your highly insightful comments! These guiding suggestions have not only enhanced this paper's academic rigor but also charted directions for subsequent research. We will proceed with:

1. Hardware-in-the-loop testing for split-μ roads and actuator fault conditions

2. Research on reinforcement learning-based multi-parameter collaborative optimization

**Reviewer#3, Concern # 5:** The manuscript proposes AEWC-SMC, while the work (Xie et al., "Highly Robust Adaptive Sliding Mode Trajectory Tracking Control of Autonomous Vehicles) develop a robust adaptive sliding mode controller. Both works contribute to the evolution of sliding mode control techniques for vehicle dynamics. The authors should potentially compare performance or highlight differences in controller design and application scenarios.

**Author response and action:** We sincerely thank you for your recognition of this paper's innovativeness and your valuable suggestions! We have carefully studied the research by Xie et al. in the field of sliding mode control ("Highly Robust Adaptive Sliding Mode Trajectory Tracking Control of Autonomous Vehicles") as recommended by you. This study proposes a modified Grey Wolf Optimizer (GWO) algorithm-optimized adaptive sliding mode controller, enhancing path tracking accuracy through vector field guidance law and intelligent optimization algorithms. This indeed shares technical relevance with our work. Following your suggestion, we have supplemented a literature comparison discussion in Section I Introduction and formally cited this reference. We reiterate our gratitude for your contribution to enhancing this paper's academic rigor! Your professional guidance serves as an important driving force for deepening this research.

**Reviewer#3, Concern # 6:** The manuscript compares its results only with traditional SMC, NTSMC, and a “No Control” scenario, omitting many recent advanced control and allocation strategies.

**Author response and action:** Thank you sincerely for your valuable feedback on the scholarly rigor of this work! In direct response to your critique regarding insufficient comparative experimentation, we have systematically redesigned simulations and analyses in Section V, now incorporating comparative experiments with both Model Predictive Control (MPC) and Fuzzy PID Control to comprehensively demonstrate performance improvements. We express our deepest gratitude for your incisive insights and constructive suggestions, which have been instrumental in elevating the quality of this manuscript and have profoundly benefited our scholarly growth.

**Reviewer#4, Concern # 1:** It is recommended to provide a more detailed explanation of the nonlinear dynamic characteristics in the 7-DOF vehicle dynamics model, such as how tire force coupling effects influence vehicle stability. The current description is somewhat abstract; adding specific formulas or case analyses could enhance logical rigor.

**Author response and action:** Thank you sincerely for your valuable feedback on the description of nonlinear dynamic characteristics in this manuscript. In response to your observation regarding the "insufficient explanation of tire force coupling effects in the 7-DOF model", we have implemented two critical enhancements in Chapter II: At the conclusion of Section II.A: Added mathematical formalization of friction circle constraints and their stability impact mechanisms. In Section II.B: Deepened the physical interpretation of weighting factors ψx and ψy. These additions have significantly improved the rigor of nonlinear characteristic descriptions through precise mathematical representation. We greatly appreciate your insightful comments, which proved fundamental to perfecting this research.

**Reviewer#4, Concern # 2:** While MATLAB/Simulink simulations are mentioned, the source of the simulation parameters (e.g., whether they are based on real vehicle data) is not clearly stated. It is advisable to supplement the parameter calibration process or reference actual test data to validate the reasonableness of the simulation conditions.

**Author response and action:** Thank you sincerely for your valuable feedback regarding the rigor of simulation parameters. All MATLAB/Simulink simulation parameters in this study were calibrated based on data from a physical vehicle test platform. The experimental platform employs a four-wheel-independent-drive electric vehicle prototype, with parameter acquisition conducted at the Intelligent Connected Vehicle Joint Laboratory co-established by Shanghai Jiao Tong University and Wuhu Advanced Research Institute. This real-world dataset will underpin subsequent experimental validation (as noted in Section VI regarding future HIL and physical vehicle testing), forming a closed-loop verification chain from simulation to physical measurement. Relevant clarifications have been added to Section V and Section VI.

We deeply appreciate your professional guidance on parameter traceability and engineering validation methodology, as well as the significant time and expertise you dedicated to enhancing the quality of this research. Your insights have substantially elevated the academic rigor of this paper.

**Reviewer#4, Concern # 3:** Although the AEWC-SMC and DWMEA methods are innovative, there is a lack of comparative analysis with existing advanced methods (e.g., reinforcement learning or deep reinforcement learning in torque distribution).

**Author response and action:** Thank you sincerely for your profound insights regarding the methodological innovation! To provide a more comprehensive demonstration of the performance improvement, we have supplemented Section V with comparative experiments involving two alternative control strategies: Model Predictive Control (MPC) and Fuzzy PID Control. Your pointed observation concerning the "lack of comparison with advanced methods like Reinforcement Learning/Deep Reinforcement Learning (RL/DRL)" demonstrates significant foresight. Although the Dynamic Weight Minimum Energy Allocation (DWMEA) approach proposed in this study possesses unique advantages in terms of non-iterative real-time capability, we fully concur that the potential of RL/DRL in adaptive weight optimization merits in-depth exploration.

Therefore, our future research will integrate Deep Reinforcement Learning frameworks (such as the DDPG or PPO algorithms) to introduce a reward function into the adaptive tuning mechanism for dynamic weight parameters. Contrastive experiments will be conducted to evaluate this approach against the closed-form solution property inherent in DWMEA.

Once again, thank you for your expert guidance. Your feedback has played a pivotal role in both refining and deepening this study. It has not only contributed to a more robust validation of performance but has also opened up crucial new directions for subsequent research.

**Reviewer#4, Concern # 4:** The simulations only compare the proposed method with traditional SMC and no-control scenarios. To more comprehensively demonstrate the performance improvements, it is recommended to add comparative experiments with other advanced methods such as MPC or fuzzy control.

**Author response and action:** Thank you sincerely for your valuable feedback on this manuscript! Your insightful suggestion to "add comparative experiments with advanced methods such as MPC or fuzzy control" provided critical direction for refining our research. In response, we have systematically incorporated detailed comparative experiments with both Model Predictive Control (MPC) and Fuzzy PID Control in Section V of the revised manuscript. These new comparative results objectively demonstrate performance improvements of our proposed control strategy across multiple performance metrics, delivering a more comprehensive validation. Your expert guidance has significantly elevated this study—not only strengthening the persuasiveness of control strategy comparisons but also pioneering valuable research directions. We deeply appreciate your rigorous review and constructive guidance, which proved instrumental in enhancing the quality and depth of this work.
